# Supplementary material for: Impact of Protein Intake on Training Response in Chronic Lung Disease
Source: Nutrients. 2025 Dec 22;18(1):41. doi: 10.3390/nu18010041 (PMC12788035; doi:10.3390/nu18010041)
Supplement: Supplementary file 1 [file nutrients-18-00041-s001.zip › Supplement S1_ProTraiL.pdf]

24-h Recall

Proband*innen ID:_______________________

Datum Interview:________________________

Tag des Recalls: ________________________Mo / Di / Mi / Do / Fr / Sa / So

Ort des Interviews:

(1) Institut für Ernährungswissenschaften
(2) Telefon
(3) Anderer Ort: ______________________________

Sind hauptsächlich Sie für die Planung und Zubereitung der Speisen in Ihrem Haushalt verantwortlich? ☐ Ja ☐ Nein

Nehmen Sie Nahrungsergänzungsmittel?

Ja ☐ Nein ☐

Liste der verzehrten Lebensmittel	Zeit	Mahlzeit	Beschreibung des LM	Menge	Ort

24-h Recall

Liste der verzehrten Lebensmittel	Zeit	Mahlzeit	Beschreibung des LM	Menge	Ort

Mahlzeit: 1. Frühstück, 2. Brunch, 3. Mittag, 4. Abendessen, 5. Spätmahlzeit, 6. Obst, 7. Pause/Snack/alkoholische Getränke, 8. Other (specify)___________________________

Bezugsort: 1. Selbst gemacht, 2. Restaurant/cafeteria/fast food shop/deli, 3. Imbiss, 4. Supermarkt, 5. Arbeitsplatz, 6. Day care, 7. Bei Freunden/Verwandten, 8. Party/BBQ/banquet/special event, 9. Other (specify)_____________________________

24-h Recall

1. Würden Sie sagen, dass die Menge, die Sie gestern gegessen verzehrt haben, normal, weniger als normal, oder mehr als normal war?

   (a) Normal (b) weniger als normal (2) (c) mehr als normal (3)

2. Was war der Grund, dass es weniger als normal war?
   a. Krankheit
   b. Geldknappheit
   c. Unterwegs
   d. Soziale Aktivität/spezielle Mahlzeit
   e. Urlaub
   f. Zu beschäftigt
   g. Keinen Hunger
   h. Auf Diät
   i. Fastenzeit
   j. Gelangweilt
   k. Gestresst
   l. Andere Gründe:____

3. Was war der Grund, dass es mehr als normal war?
   a. Unterwegs
   b. Soziale Aktivität/spezielle Mahlzeit
   c. Sehr hungrig
   d. Gelangweilt
   e. Gestresst
   f. Andere Gründe:____

4. Wie würden Sie Ihr aktuelles Essverhalten beschreiben?
   a. Keine spezielle Diät, ich esse fast alles
   b. Vegetarier
   c. Veganer
   d. Anderes:____
   ____

Vielen Dank!

Bemerkungen:
