# Supplementary material for: Impact of Protein Intake on Training Response in Chronic Lung Disease
Source: Nutrients. 2025 Dec 22;18(1):41. doi: 10.3390/nu18010041 (PMC12788035; doi:10.3390/nu18010041)
Supplement: Supplementary file 1 [file nutrients-18-00041-s001.zip › Supplement S2_ProTraiL.pdf]

Merkblatt Protein

In der zweiten und dritten Phase der „ProTraiL-Studie“ werden Sie gebeten, ihre täglichen Ernährungsgewohnheiten zu verändern.

Bitte achten Sie selbstständig darauf, Ihren persönlichen Eiweißbedarf über ihre alltägliche Ernährung zu erreichen.

Das angehängte Dokument soll Ihnen dabei helfen, Ihren Eiweißbedarf über die normale Ernährung zu decken.

Bitte nutzen Sie dazu bevorzugt unverarbeitete Lebensmittel. Es ist nicht notwendig, auf Produkte zuzugreifen, die mit einem hohen Proteinbedarf werben. Insbesondere Lebensmittel mit Werbeslogans wie „High Protein“, „Eiweißbrot“ oder Fertigtiefkühlprodukte sind häufig hochverarbeitet und tragen **nicht** zu einer gesunden, proteinreichen Ernährung bei.

Vielen Dank für Ihre Teilnahme!

Ihr persönlicher täglicher Eiweißbedarf:

Institut für Ernährungsmedizin
Klinikum rechts der Isar, TU München
Uptown München Campus D
Georg-Brauchle-Ring 60/62 München

Direktor: Univ.-Prof. Dr. Hans Hauner

Eiweißaustauschtabelle

<u>10 g Gramm Eiweiß sind enthalten in:</u>

Milch und Milchprodukte
280ml Buttermilch
300ml Kuhmilch, Joghurt, Dickmilch, Kefir (3,5% F)
280ml Kuhmilch, Joghurt, Kefir (0,1% F)
325g Saure Sahne (10% F)
380g Schmand (24% F)
420ml Sahne (30% F)
500g Creme Fráiche (40% F)

Käse und Ei
35g: Emmentaler (45% F.i.Tr.), Harzer Korbkäse (ca. 1% F)
Gruyére (48% F.i.Tr.), Bergkäse (45% F.i.Tr.)
40g: Appenzeller (50% F.i.Tr.), Edamer (45% F.i.Tr.),
Gouda (40% F.i.Tr.), Tilsiter (45% F.i.Tr.), Raclette-Käse
45g: Brie (50% Fi.Tr.), Camembert (30% F.i.Tr.)
50g: Camembert (45% Fi.Tr.), Gorgonzola, Mozzarella
55g: Camembert (60%F.i.Tr.), Butterkäse (60% F.i.Tr.)
70g: Schmelzkäse (45% F.i.Tr.), Speisequark mager,
Hüttenkäse
90g: Speisequark (40% F.i.Tr.),
Doppelrahmfrischkäse (60% F.i.Tr.)
110g: Mascarpone

80g: Vollei (entspricht 11/2 Eier der Grösse M)

Fette und Öle
Speiseöle enthalten kein Eiweiß.
Der Eiweißgehalt in Butter, Margarine oder Schmalz liegt unter 0,5g/100g Lebensmittel und muss somit nicht berücksichtigt werden.

Gemüse und Pilze
Alle Gemüsesorten, Pilze und Kräuter sind relativ eiweißarm und enthalten im Durchschnitt nur 2-3g Eiweiß/100g, sodass eine Portion von 200g selten mehr als 5 Gramm Eiweiß enthält. Hülsenfrüchte und Soja (Tofu) sind trotz ihres höheren Eiweißgehaltes geeignet, da sie pflanzliches Eiweiß liefern.

Obst und Obstprodukte
Alle Obstsorten, Obstsäfte und Konfitüren sind eiweißarm und enthalten in der Regel nicht mehr als 0,3 bis 3g Eiweiß pro 100g. Sie müssen bei der Eiweißberechnung nicht berücksichtigt werden.

Diverses
Der Eiweißgehalt in Ketchup, Mayonnaise, Remoulade, Gewürzsoßen oder Meerrettich kann vernachlässigt werden.
Auch vegetarische Brotaufstriche helfen Eiweiß sparen, wenn sie alternativ zu Wurst und Käse verwendet werden.

Quelle: “Die große GU Nährwert Tabelle”
Ausgabe 2014/2015

10 g Gramm Eiweiß sind enthalten in:

Fisch und Fischwaren
50g: Forelle, Zander
55g: Kabeljau, Rotbarsch, Scholle
60g: Krabben, Hering
90g: Muscheln
35g: Lachs, geräuchert
45g: Thunfisch in Öl, Ölsardinen
50g: Makrele, geräuchert
55g: Aal, geräuchert
60g: Matjesfilet, Brathering
70g: Hering in Tomatensoße

Geflügel
40g: Putenbrust
50g: Brathuhn
55g: Hähnchenkeule
55g: Ente
65g: Gans

Fleisch und Wild
45g: mageres Fleisch von Schwein, Rind
50g: mageres Kalbfleisch
55g: Hammelfleisch
50g: Hackfleisch, gemischt
45g: Reh, Hase
50g: Hirsch, Kaninchen

10 g Eiweiß sind enthalten in:

Wurst
45g: Schinken (ohne Fettrand)
55g: Salami
60g: Bierschinken, Leberwurst
70g: Mettwurst
80g: Fleischkäse, Leberkäse, Mortadella
90g: Münchner Weißwurst
100g:Gelbwurst, Fleischwurst, Wiener Würstchen

Brot und Getreide
130g: Brot im Durchschnitt
100g: Knäckebrot, Zwieback
80g: Nudeln, eifrei
90g: Grünkern, Dinkel
100g: Hirse
140g: Reis
100g: Weizenmehl, Typ 405
100g: Weizengrieß

Hülsenfrüchte, Nüsse und Samen
50g: Hülsenfrüchte i.D. getrocknet / 150-200g gekocht
120g:Tofu
40g: Erdnüsse, Leinsamen, Sonnenblumenkerne geschält
60g: Cashewnüsse, Sesam
70g: Walnüsse, Haselnüsse
